# Differential effects of negative emotion on memory for items and associations, and their relationship to intrusive imagery

**DOI:** 10.1016/j.cobeha.2017.07.012

**Published:** 2017-10

**Authors:** JA Bisby, N Burgess

**Affiliations:** 1 Institute of Cognitive Neuroscience, University College London, London, UK; 2 Institute of Neurology, University College London, London, UK

## Abstract

A crucial aspect of episodic memory formation is the way in which our experiences are stored within a coherent spatio-temporal context. We review research that highlights how the experience of a negative event can alter memory encoding in a complex manner, strengthening negative items but weakening associations with other items and the surrounding context. Recent evidence suggests that these opposing effects can occur through amygdala up-modulation to facilitate item encoding, while the hippocampal provision of contextual binding is down-modulated. We consider how these characteristics of memory for negative events might contribute to the development and maintenance of distressing intrusive imagery in posttraumatic stress disorder, and how they should influence therapeutic interventions.

## Highlights

• Negative emotion can affect memory for items and associations differentially.
• Strengthened item memory reflected in increased amygdala activity.
• Weakened contextual/associative memory reflected in reduced hippocampal activity.
• Imbalance between strong negative items and weak contextual associations predicts intrusions.

**Current Opinion in Behavioral Sciences** 2017, **17**:124–132

This review comes from a themed issue on **Memory in time and space**

Edited by **Lila Davachi** and **Neil Burgess**

For a complete overview see the Issue and the Editorial

Available online 21st September 2017

**http://dx.doi.org/10.1016/j.cobeha.2017.07.012**

2352-1546/© 2017 The Authors. Published by Elsevier Ltd. This is an open access article under the CC BY license (http://creativecommons.org/licenses/by/4.0/).

## Introduction

Experiencing a negative event, such as the aftermath of a motor accident, can severely impact memory [1] and in some situations can result in debilitating memory disturbances, as observed in posttraumatic stress disorder (PTSD) [2]. Understanding how negative emotion interacts with memory is therefore important for informing treatment. A fundamental aspect of episodic memory formation is that experiences are stored within a coherent spatio-temporal context, allowing for their flexible and holistic retrieval [3]. This requires not only storing the items and content of an event but also binding those elements to each other and the surrounding context.

Although it is often assumed that negative emotional content will generally strengthen memory [4], an alternative view proposes that memory for the negative content of an event will be enhanced by boosting amygdala activity, but that hippocampal processing to bind together the items and context comprising the event will be weakened [5, 6••]. Here, we discuss the mechanisms by which negative emotion can have opposing effects on memory encoding for items and associations, and the neural structures that support them. Further, we discuss how the resulting imbalance in memory might give rise to the intrusive imagery that can occur in PTSD.

## Memory for items and associations

Several models dissociate memory for the items within an event and the associations between them and the surrounding context [7, 8, 9, 10]. Within this review, we refer to the content specific to a given event such as an object or a person (or a word in laboratory experiments) as items, as compared to the associations or relations between multiple items or the items and their surrounding context. Importantly, these associations enable the formation of coherent memory representations, from which items, context and the relations between them can be freely recalled, whilst the item representations alone can only support item recognition and familiarity judgments.

Within this conceptualization, the hippocampus is thought to act as a convergence zone, binding together multi-modal information into a single coherent representation [11, 12, 13, 14], and giving rise to the holistic multi-modal recollective experience through reinstatement of all associated information via pattern completion [11, 13], a hallmark characteristic of episodic memory [7, 8, 10]. In contrast, item or familiarity-based recognition is thought to be supported by structures outside of the hippocampus [7, 8, 15]. For example, perirhinal and parahippocampal areas might store domain-specific representations of items and scenes, respectively, which the hippocampus binds together into a coherent domain-general representation [16, 17, 18] to support their reinstatement and holistic retrieval [19, 20]. Given the increasing evidence of partially differentiated systems for remembering single items from an event versus how they are associated with each other or with the surrounding context, it is vital to establish whether emotional experiences interact differentially with these systems, and if so, how these differences affect the experience of memory.

## Opposing effects of negative emotion on items and associations

Behavioral evidence supports the view that memory for negative items themselves is strengthened, but the associations with other items and with the appropriate context is disrupted. Strengthened item memory for negative words or images has reliably been shown when compared to matching neutral stimuli [21]. This enhancement is evident across a broad range of measures including accuracy, confidence, vividness [22, 23] and a subjective sense of recollection [24]. However, the recollective experience often attributed to negative events is specific to the negative content itself and does not correspond to improved memory for associated contextual details [25, 26].

**Figure 1:**
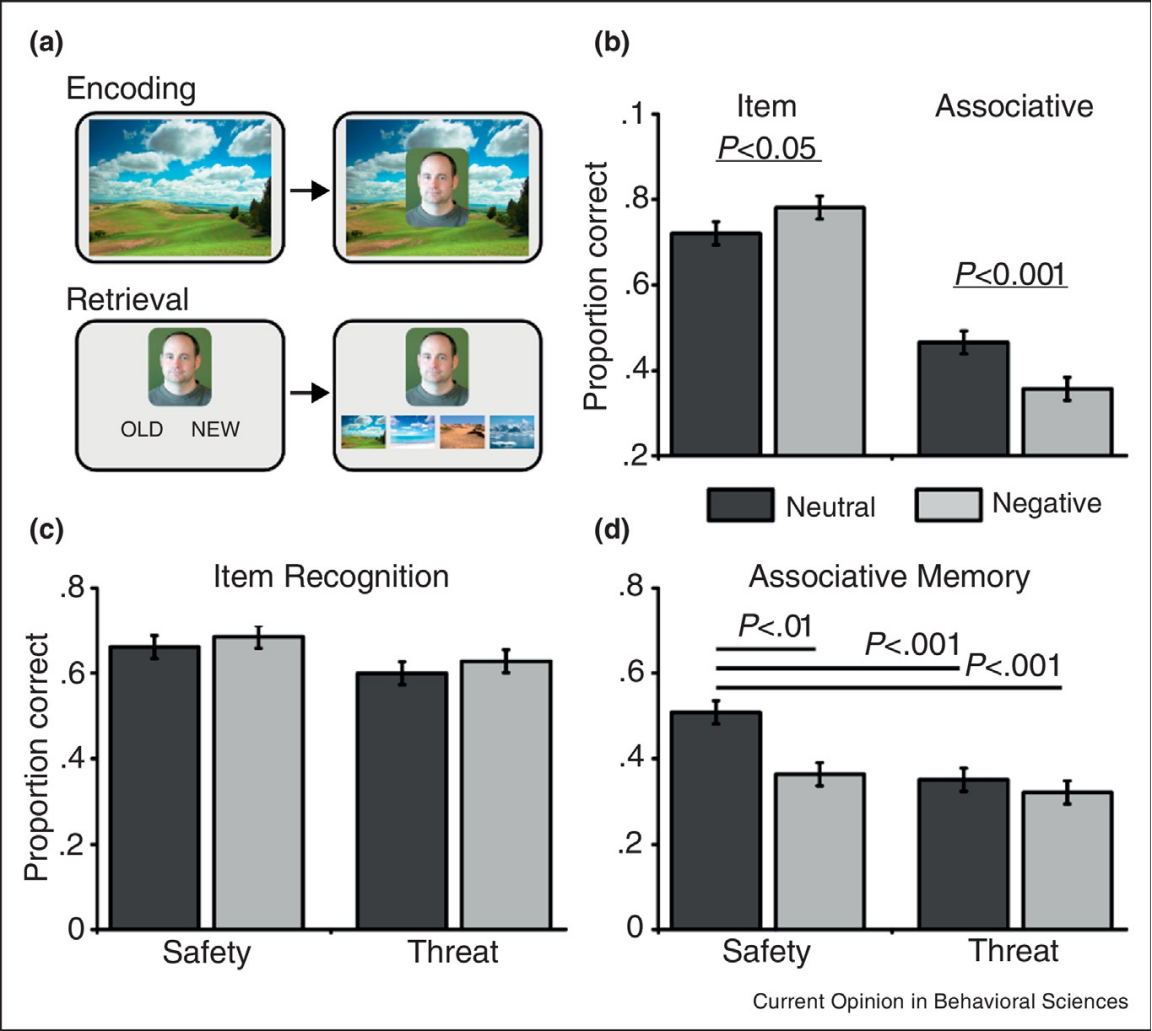
**(a)** To assess, item and item–context memory, participants studied neutral and negative items presented on neutral background contexts. Memory was tested for the items and the associations between the items and their context. **(b)** Whilst memory for negative items is enhanced, memory for associations between the negative items and their neutral context is impaired. **(c and d)** To rule out the effect of attentional capture by the negative item itself, participants were instructed that certain contexts were predictive of a mild electrical shock. Whilst recognition memory for neutral and negative items was unaffected by safe and threatening contexts, associative memory for the context of neutral items was disrupted in the threatening context [27].

In contrast to item memory enhancements, negative emotion can disrupt memory for forming associations between different items, or between items and the associated spatial context. For example, following the encoding of item–context pairs, participants demonstrate worse associative accuracy for identifying the context in which negative items appeared, despite improved memory for the negative content (see Figure 1a,b) [27]. Numerous studies have demonstrated robust impairments using a wide range of tasks, including recognizing peripheral details (whether an object or the color of a framing border) when presented in combination with negative items [28, 29, 30], associating negative words [31] or images [27, 32•] or associating the items with their context [30].

Further evidence demonstrates how negative emotion can disrupt the coherence of memories for events. In a recent study [33^•^], participants studied events comprising person-location-objects triplets, with memory later tested for all pairs from each event to assess the dependency between retrievals from the same event (e.g. cueing with the person to retrieve the associated location). Results showed that when one of the items from an event was negative (e.g. an injured person), memory coherence was reduced (less dependency between retrieval trials from the same event) compared to neutral events (which show holistic retrieval related to hippocampal processing [19]). Overall, this study highlights how the presence of a negative item in an event can disrupt within-event associations leading to reduced memory coherence and a fragmented representation.

Whilst many of the described studies highlight the complex ways in which negative emotional content can impair memory, we propose that impairments are seen when associative memory processes are required for successful performance. Of note, memory for the screen location of an item is often improved for negative items [29, 34, 35]. We assume that recognition of an item's location relative to the perceiver might not be bound to other items or the spatial context (see below for further discussion on egocentric representations of negative items), but is a characteristic that could be stored as part of the item's perceptual representation.

Of course, the salience of emotional items will attract greater processing due to their attentional capture and distinctiveness [36], explaining many of the positive effects of emotion on item encoding [37]. However, the attraction of attention toward negative items cannot fully account for disruptions in associative memory. For example, when participants encode neutral and negative item–context pairs presented on background contexts predicting either safety or a threat of a mild electric shock, the threatening contexts had little effect on item memory, but impaired association of the neutral item with its context (see Figure 1) [27]. Further, when participants encode negative–negative item pairs, reducing their relative distinctiveness and attentional competition, associative memory is still disrupted compared to neutral–neutral item pairs [27, 32•].

## The amygdala and enhanced item memory

The amygdala has emerged as a prime candidate for orchestrating memory enhancements for negative items. The amygdala is thought to facilitate attentional processing and encoding of negative items through up-modulation of sensory areas [38] and memory-related medial temporal lobe structures [39]. Indeed, amygdala activity during encoding of negative items is predictive of subsequent memory performance [21], and its activity is further increased by adopting a more perceptual processing strategy [40]. Memory enhancements for emotional but not neutral items are attenuated or abolished in patients with damage to the amygdala, supporting a modulatory influence on other medial temporal lobe (MTL) structures [41].

Although memory enhancements for negative items might reflect amygdala-driven up-modulation of MTL structures, the hippocampus may not be the main target, as patients with selective hippocampal damage demonstrate enhanced memory for negative items when tested following a short delay [41, 42]. In one study, two patients with bilateral hippocampal damage were asked to recognize neutral and negative images, tested using remember-know judgements [24]. Interestingly, whilst recollection of recognized images was reduced in amnesics, an emotional enhancement was still observed in familiarity-based recognition. Thus, item memory enhancements for negative content can occur without hippocampal provision of contextual support.

The selective memory enhancement for negative items seen without the hippocampus suggests that the amygdala interacts with other MTL regions to facilitate item encoding [18]. For example, it could modulate memory by reinforcing the emotional properties of an item [43, 44•] via its dense connections with perirhinal cortex [45], consistent with its role in fear conditioning [46]. Neuroimaging studies have shown both greater perirhinal activity [32^•^] and increased functional connectivity with the amygdala [47] during encoding of subsequently remembered negative items, and evidence suggests that the amygdala plays a more general role in supporting item memory irrespective of emotion [32•, 48, 49, 50]. Further, the amygdala plays a role in memory consolidation and, thus, might interact with perirhinal cortex to strengthen the binding of the experienced emotion to the specific items while hippocampal-dependent associations are forgotten more rapidly [18, 44•].

## The hippocampus and reduced associative memory

**Figure 2:**
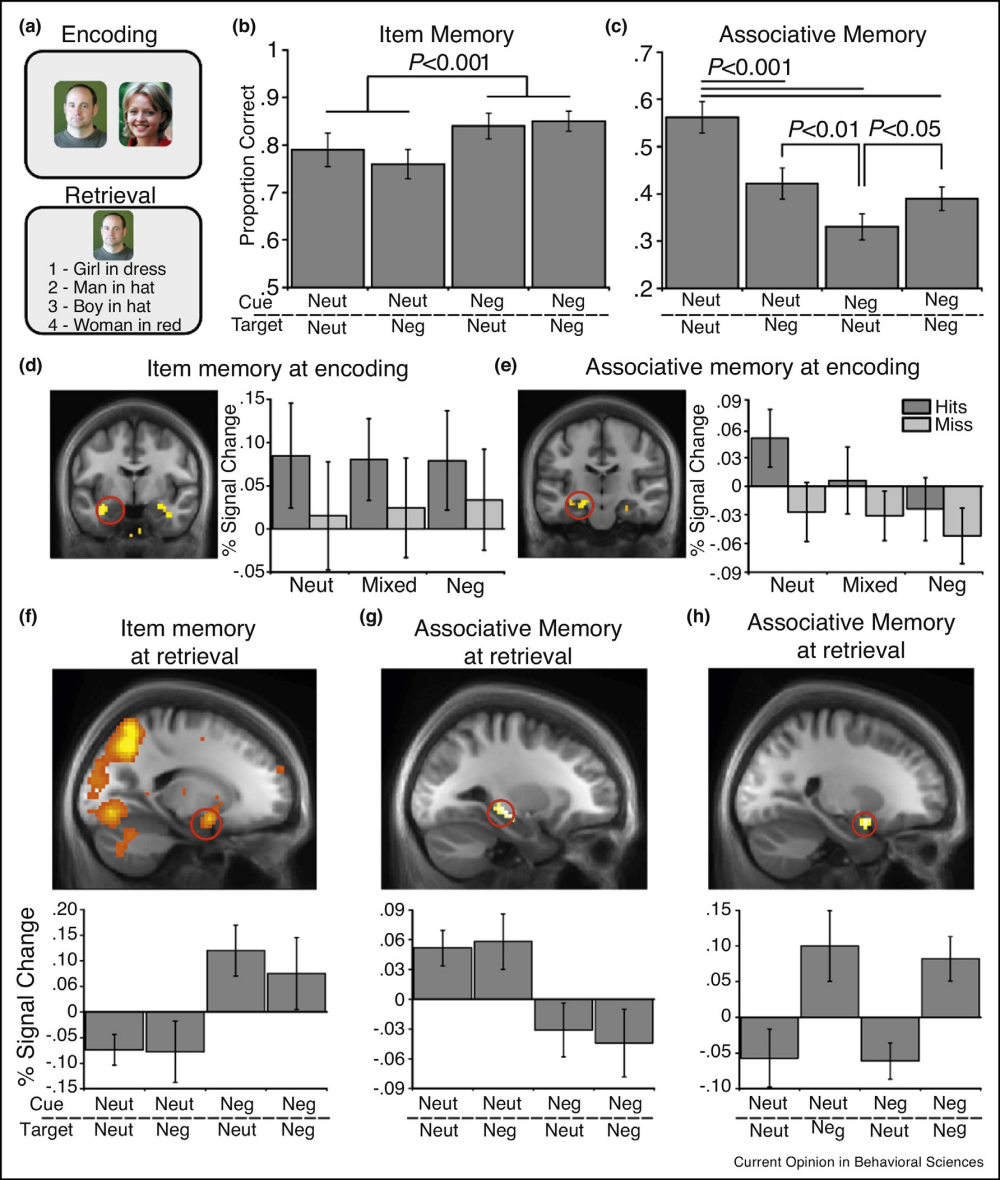
**(a)** Participants encoded paired associates of neutral and negative images. Memory was tested by presenting one item from each pair and asking participants if they recognized the item and if so, to identify the image it was paired with from a list of descriptions. **(b)** Recognition of negative item cues was better than for neutral items. **(c)** Associative memory was reduced by the presence of negative items at encoding. **(d)** Increased amygdala activity at encoding was predictive of subsequent item memory, whereas the hippocampus supported associative memory **(e)**. Hippocampal activity was reduced by the presence of negative items at encoding. **(f)** At retrieval, increases in amygdala activity predicted negative item recognition. **(g)** The presence of a negative item cue reduced hippocampal activity during associative retrieval. **(h)** Amygdala activity increased when retrieving a negative associate from memory even when the cue was neutral, corresponding to a boost in retrieval of negative associates (c) [32^•^].

What changes in neural processing might prevent a negative experience from being stored within a coherent representation with intact associations between items and context? A recent fMRI study suggests that the presence of negative items disrupts the ability of the hippocampus to bind together multi-modal information (see Figure 2) [32^•^]. Participants encoded paired associates constructed from all combinations of neutral and negative images. At test, they were cued with one image from a pair and tested for recognition of the item and memory for the associated image. As in previous studies, memory for negative items was enhanced whereas memory for the associations between them was reduced when a negative item was present (even if both items were negative). At encoding, amygdala activity was predictive of subsequent item memory, while increased hippocampal activity predicted successful associative memory (see Figure 2b), see also [51]. However, hippocampal activity decreased in the presence of negative items, corresponding with reduced associative memory. Interestingly, successful retrieval of a negative associate from memory was related to increases in amygdala activity, even when a negative item was not present on the screen.

Further studies support the idea that negative emotion disrupts hippocampal-dependent associative memory but not memory for negative items themselves. In one fMRI study, associative memory for face–occupation pairs was reduced when faces were paired with a negative occupation and hippocampal activity was correspondingly reduced during their encoding [52]. In another study, reduced hippocampal activity was observed after watching negative videos and this reduction correlated with better memory for negative items seen immediately following the videos [53]. Thus, the experience of the negative event could alter processing by focusing resources to process the salient threatening or negative information at the expense of hippocampal-dependent associative processing.

The exact mechanisms that might cause hippocampal down-modulation in the presence of negative items is unclear. Increases in stress can disrupt hippocampal function and memory [54]. The stress response typically slow, rapid effects of impaired item–context memory can be observed when individuals are administered cortisol prior to encoding [55]. Equally, interactions between glutamate and noradrenaline during emotional encoding that could upregulate high priority information, whilst impairing lower priority representations [56], and hippocampal down-modulation could be mediated by mPFC connections with inhibitory neurons in the hippocampus via the nucleus reunions [57]. Interestingly, directed forgetting can occur via hippocampal downregulation at encoding, possibly through top-down inhibition from lateral prefrontal areas [58].

## Implications for intrusive imagery

**Figure 3:**
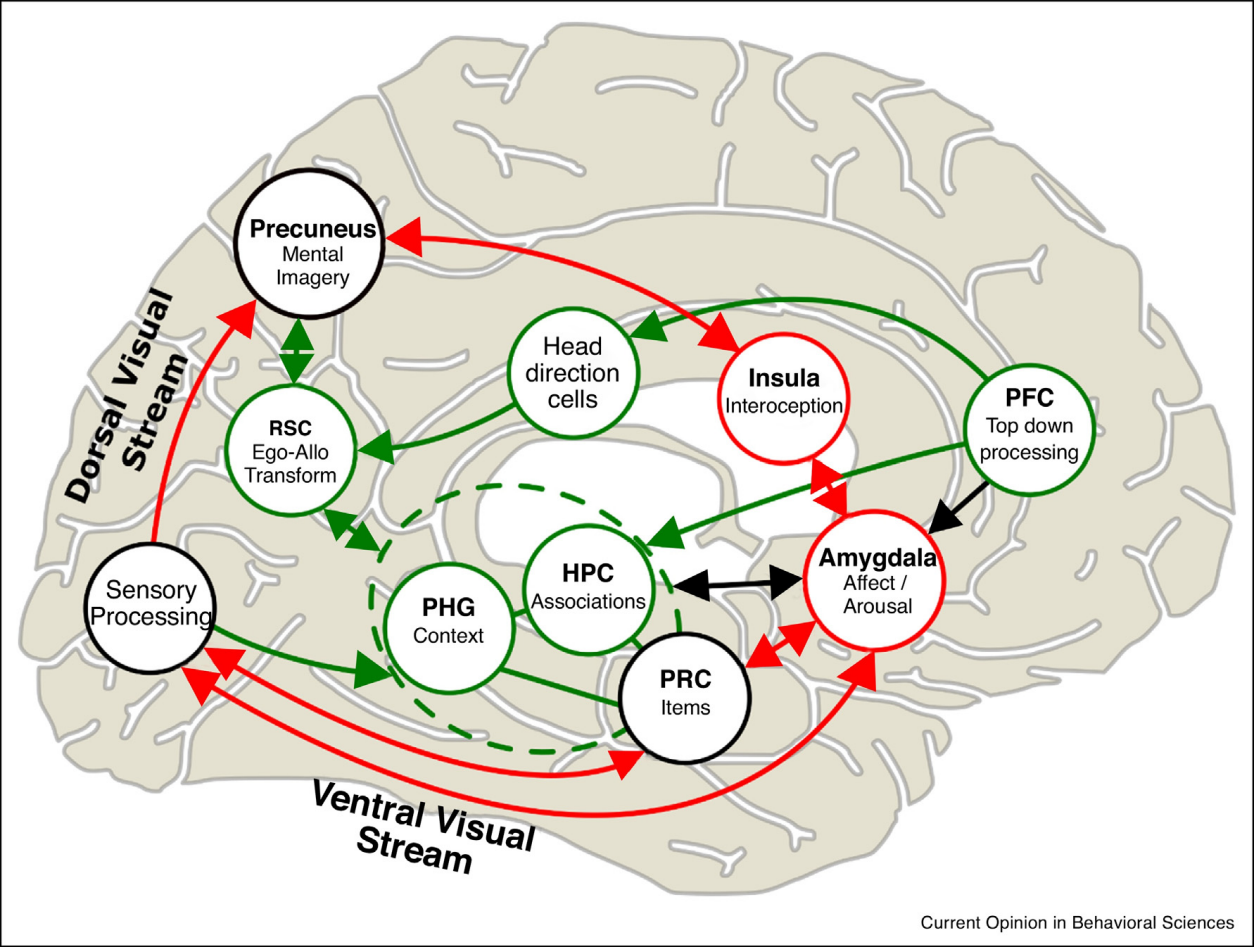
Schematic dual representation model. **Green lines**: In memory for neutral events items represented in perirhinal cortex (PRC) are bound together with the corresponding context (parahippocampal gyrus, PHG) in the hippocampus (HPC). Voluntary retrieval can be initiated by ‘top-down’ input from prefrontal cortex (PFC), reconstructing an allocentric representation of the scene of the event via pattern completion in the medial temporal lobe (MTL, dashed green line), translated via retrosplenial cortex (RSC) to produce egocentric imagery in the Precuneus (following [59•, 60•]). The resulting scene is consistent with the viewpoint indicated by HPC place cells and the view direction indicated by head-direction cells. **Red lines**: For traumatic events, strong sensory/affective item representations are also formed in the Insula, PRC and Sensory areas via processing in the amygdala. In healthy memory, voluntary retrieval of a traumatic event occurs via the hippocampal system under control from PFC (green lines). However, following a traumatic event, intrusive imagery may occur due to an imbalance between strongly represented negative content, boosted by the amygdala (red lines), and contextual representations rendered weak and fragmented by reduced associative processing in the rest of the MTL (green dashed lines). This allows reactivation of negative content to be triggered via environmental cues and experienced as distressing imagery in precuneus, outside of its associated context [6^••^].

A core symptom of PTSD is the persistent occurrence of distressing involuntary imagery of the negative content from a traumatic event [2]. Within the spirit of this review, we consider intrusive imagery to include the negative content from the real-life trauma, comprising separable items such as the knife or the face of an approaching attacker. Again, we assume that associations between these negative aspects of the event and the surrounding context and neutral items are also required to create a coherent representation in episodic memory. Within this conceptualization and the empirical evidence we have outlined, healthy memory (see Figure 3) comprises both representations of the sensory and affective qualities of items supported by sensory areas and amygdala and associated contextual and neutral representations supported by the hippocampus. Deliberate recollection can be driven by ‘top-down’ inputs from prefrontal cortex triggering pattern completion in hippocampus and thus controlled reactivation of contextual and item representations.

A dual representation account proposes that a traumatic experience will up-modulate the amygdala to strengthen encoding and storage of the negative content, whereas disruption of hippocampal processing will lead to weakened associations between items and the experienced context (see Figure 3) [5, 6••]. The resulting imbalance, of a strong enduring representation for the traumatic items with a weakened contextual representation, creates a persistent emotion-laden image of the traumatic content lacking associations with other neutral content and the surrounding context. Voluntary retrieval will be reduced via a lack of hippocampal support, but sensory cues in the environment with similarity to the original traumatic content can trigger involuntary retrieval of distressing images that are re-experienced out of context.

## Modulating intrusive memories

Many aspects of the development of intrusive imagery can be understood in terms of the incidental triggering of sensory/affective representations. A dual representation account proposes a distinction between representations of the negative items of the event (strengthening of which should *increase* intrusions, as noted above) and hippocampal-dependent representations of the associations between items and the context of the person experiencing the event (strengthening of which should *decrease* intrusions) [61]. Thus, disrupting visuospatial processing during encoding [62] or consolidation of traumatic video footage can decrease the frequency of intrusive memories experienced subsequently, by weakening the pathway between potential visuospatial triggers and the negative content [63]. Conversely, providing more information about a negative event may increase the number of potential retrieval cues [64], as might ongoing re-activation or rumination concerning negative events [65], increasing the frequency of subsequent intrusive thoughts. Supporting this view, participants with PTSD show enhanced recognition of perceptually degraded trauma-related pictures [66].

A study using alcohol as a pharmacological tool, due to its ability to impair hippocampal-dependent memory [67], provides direct support for a dual representation account of intrusive imagery [68]. Participants consumed either placebo or a low or high dose of alcohol prior to watching video clips showing traumatic events. Participants also completed an object-location task in which memory was tested from the same-view or a shifted-view, to examine the balance between egocentric and allocentric representations of spatial location, since the latter but not the former had been found to be hippocampal dependent [69]. Within this task, object-locations can be recognized from the same-view as encoding on the basis of egocentric sensory representations, whereas shifted-view accuracy requires an allocentric representation of the wider spatial context [69], a hallmark characteristic of episodic retrieval [3]. Results showed that a low dose of alcohol during traumatic footage increased the number of intrusive memories reported in the following week. Further, the low dose of alcohol selectively impaired object–location performance when tested from a shifted-view but not from the same view. Importantly, the imbalance between reduced allocentric memory relative to preserved egocentric memory correlated with the number of intrusive memory experienced in the low dose group. Consistent with the view that contextual associations can reduce intrusions, a recent study showed that faster recognition of intact item–context pairs correlates with reduced intrusive memory reports for traumatic video footage [70]. Finally, participants with PTSD have been shown to be specifically impaired at allocentric spatial processing [71, 72] and to have reduced hippocampal volume [73].

## Therapeutic implications

Disruptions in forming associations between the traumatic content of an event and its context that contribute to intrusive imagery may well have implications for PTSD. Facilitating associations between the negative event and the context in which it took place, or with novel contexts, might help to reduce memory disruptions [6^••^]. Trauma-focused therapies, such as eye movement desensitization and reprocessing (EMDR) and imagery re-scripting techniques, aim to alleviate ongoing symptoms by revisiting and elaborating on the traumatic episode while maintaining a distance between the previously experienced event and the current context. These techniques could help to build new associations between the traumatic material and their contextual support to place the episode in a coherent past memory representation that can reduce involuntary imagery. It may be that strong association with hippocampal contextual representations allows recollection of traumatic material to be controlled by top-down inhibition from prefrontal areas [58] which acts via the inhibition of the hippocampus [74] but cannot directly inhibit traumatic sensory representations.

## Conclusions

We have highlighted some of the potential complexities inherent to the interaction between negative emotion and memory. Whilst the prevailing view has often been that negative emotion will strengthen memory [23], with this effect possibly also modulated by attention [36], we reviewed evidence that different aspects of memory have different interactions with negative emotion. Thus, while the negative items of an event might be enhanced through amygdala up-modulation, the presence of negative affect might disrupt normal hippocampal function and the associative binding of items and their appropriate context, resulting in a fragmented representation lacking top-down control. Although based on findings in healthy volunteers, this evidence supports the basic mechanism suggested by dual representation accounts of PTSD [5, 6••], suggesting that observed memory disruptions in PTSD and memory intrusions result in part from an imbalance between strengthened representations of the emotional content but weakened associations to other neutral items and contextual information. Future studies in clinical populations will be required to test whether this interpretation of PTSD is useful for understanding and treating this widespread and debilitating condition.

## Conflict of interest statement

Nothing declared.

## References and recommended reading

Papers of particular interest, published within the period of review, have been highlighted as:

• of special interest
•• of outstanding interest
